# Case Report: Pancreatic Neuroendocrine Tumor With Liver Metastasis and Portal Vein Thrombosis

**DOI:** 10.3389/fonc.2021.809129

**Published:** 2022-02-14

**Authors:** Yulong Tian, Xingshun Qi, Afaf Aljbri, Ke Xu, Hongshan Zhong

**Affiliations:** ^1^ Department of Interventional radiology, the First Hospital of China Medical University, Shenyang, China; ^2^ Department of Gastroenterology, General Hospital of Northern Theater Command, Shenyang, China

**Keywords:** pancreatic neuroendocrine tumor, liver metastases, portal vein, interventional therapy, anticoagulant therapy

## Abstract

**Introduction:**

Pancreatic neuroendocrine neoplasms (PNENs) are rare pancreatic tumors originating from pancreatic neuroendocrine cells. There is no consensus on the treatment for PNENs with unresectable liver metastases. Transcatheter arterial chemoembolization (TACE) is the preferred treatment for unresectable primary liver cancer. But the efficacy of TACE and anticoagulation in PNENs with unresectable liver metastases and portal vein thrombosis has never been reported.

**Methods and Results:**

We present the case of a 50-year-old male patient with hepatitis C who was found to have a single liver mass during a regular physical examination in 2016. The liver mass was surgically removed. Postoperative pathology suggested a neuroendocrine tumor of the liver, and it was suggested to look for the primary tumor. The patient was followed up until 2020, and the primary pancreatic tumor was found, along with multiple liver metastases and portal vein thrombosis. After transcatheter arterial embolization, anticoagulation, and endocrine therapy, the patient’s tumor load was relieved, and the portal vein was recanalized.

**Conclusion:**

The article reports the disease course in a case of a functional pancreatic neuroendocrine tumor with liver metastasis and portal vein thrombosis and reviews previous literature. To our knowledge, we reported for the first time the efficacy of TACE and anticoagulation in PNENs with unresectable liver metastases and portal vein thrombosis.

## Case Presentation

**Figure 1:**
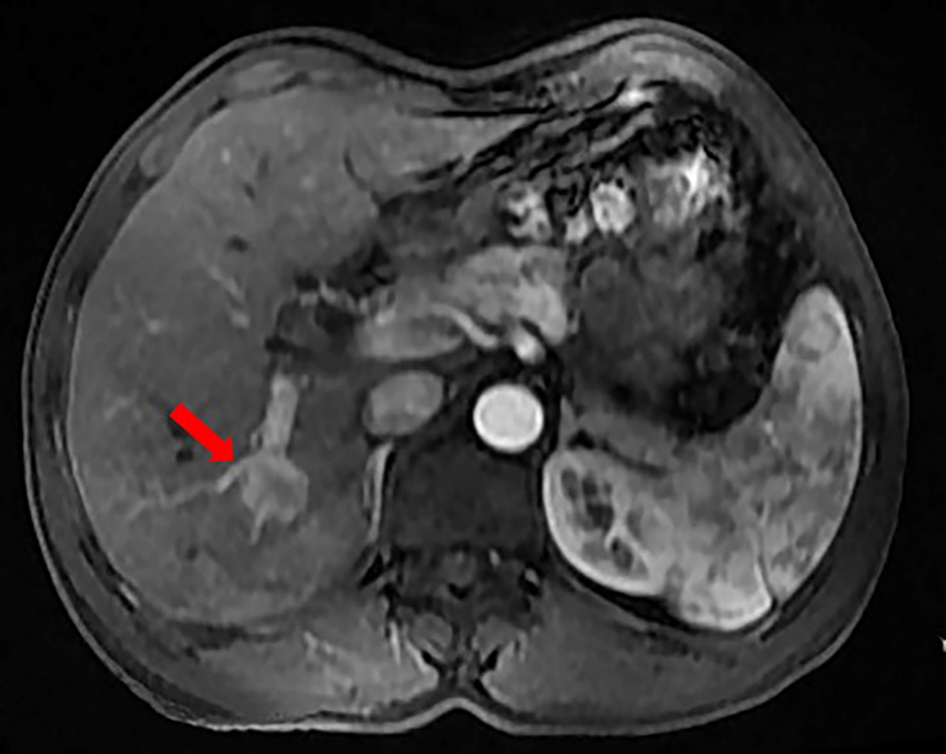
The patient was found to have a single liver mass during a regular physical examination in 2016.

**Figure 2:**
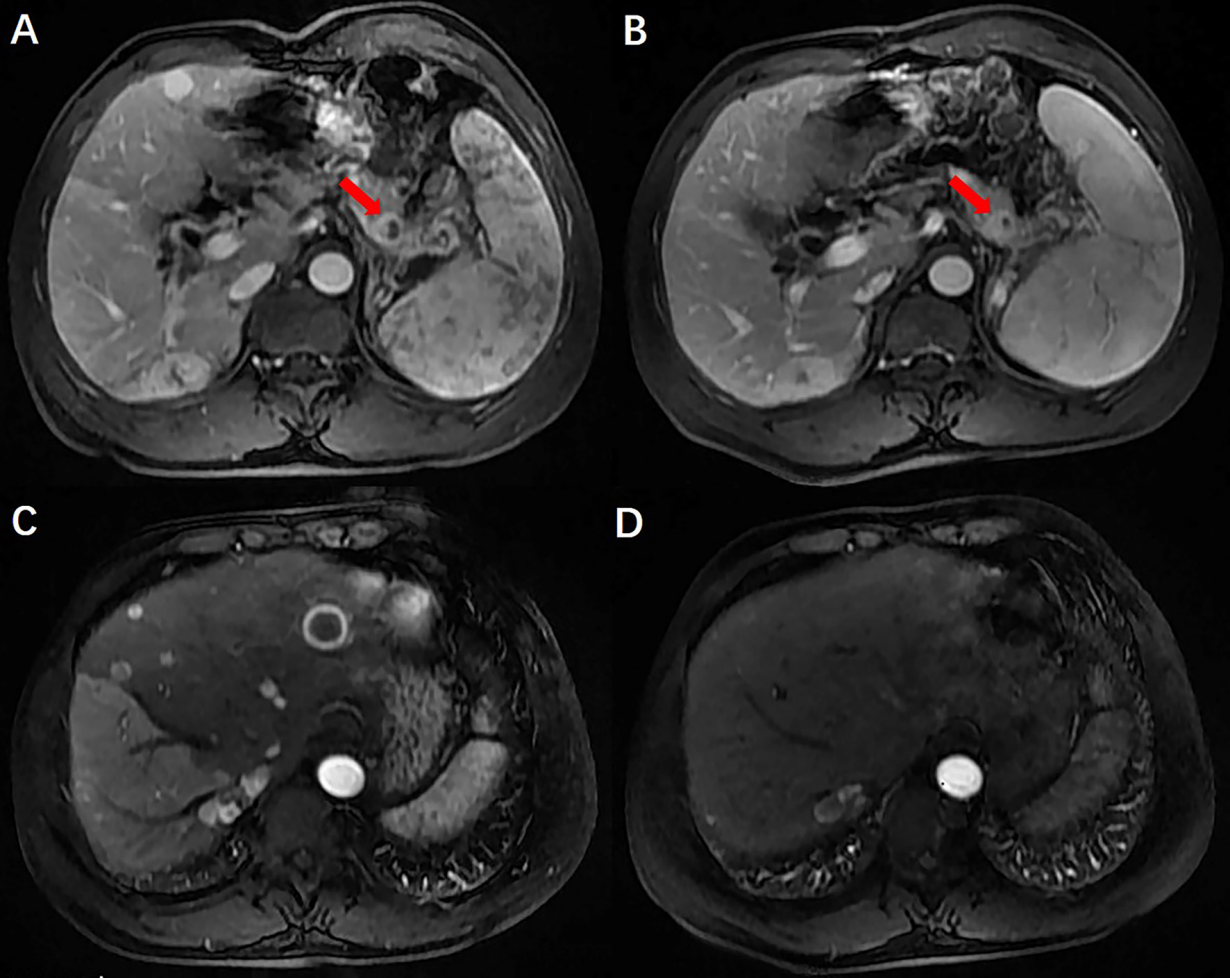
Preoperative and postoperative comparison of PNENs and liver metastases. There was no change in the size of the pancreatic lesion and intrahepatic tumor of the patient was reduced.

**Figure 3:**
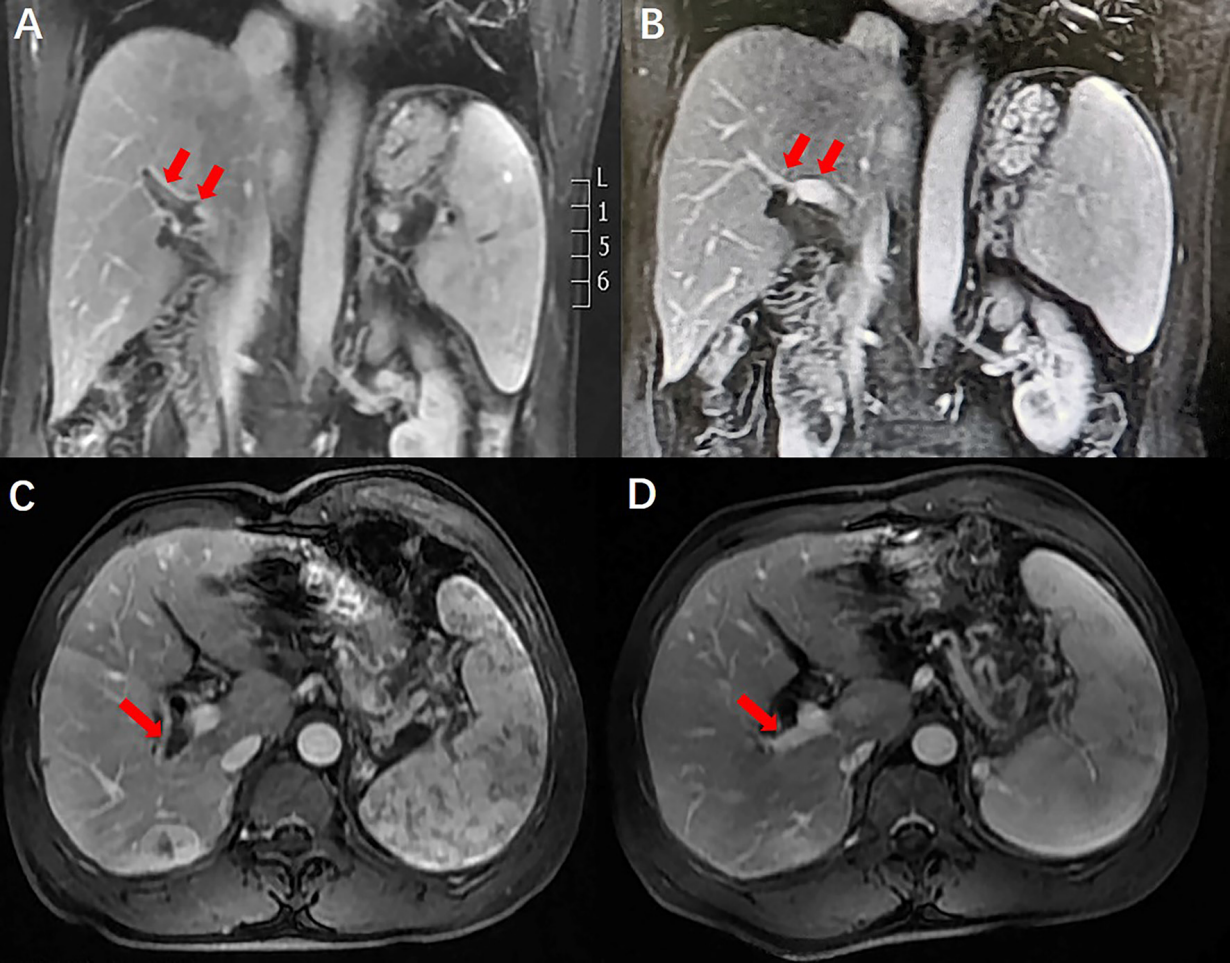
Comparison of portal vein thrombosis before and after anticoagulant therapy. Portal vein thrombosis was recanalization.

A 50-year-old man with hepatitis C was found to have a single liver mass during a regular physical examination in 2016 (**Figure 1**). MRI scan of the abdomen demonstrated a 21 mm × 17 mm hypervascular tumor in the lower right posterior lobe of the liver. The patient underwent a right lobe hepatectomy. The histopathological findings of the specimen revealed a synaptophysin-positive and chromogranin-A-positive neuroendocrine neoplasm. Pathological findings further excluded liver metastasis from gastrointestinal and pancreatic neoplasms. But no evidence of a primary tumor was found. The patient recovered well after surgery and was followed up every year. Primary pancreatic mass was observed in 2020 (**Figure 2A**). Meanwhile, MRI revealed multiple hepatic metastases (**Figure 2C**) and portal vein and splenic vein thromboses (**Figures 3A, C**). There was an abnormal intrahepatic blood supply in the right lobe of the liver. The patient underwent hepatic artery embolization. Two embolism microspheres were used during the interventional procedure (d = 100–300 μm). After that, octreotide acetate microspheres for injection (Shanlong^®^, 30 mg) was used; at the same time, subcutaneous injection of low molecular heparin calcium 0.4 ml was given twice a day for a total of 3 days. Rivaroxaban 20 mg was taken orally for 1 month. Follow-up results showed there was no change in the size of the pancreatic lesion (**Figure 2B**). Intrahepatic tumor of the patient was reduced (**Figure 2D**), and recanalization of portal vein thrombosis (**Figures 3B, D**) and partial recanalization of splenic vein thrombosis were performed. Abnormal intrahepatic blood supply was recovered well.

## Discussion

### Pancreatic Neuroendocrine Neoplasms

#### Clinical Characteristics

Pancreatic neuroendocrine neoplasms (PNENs) are rare tumors of the pancreas and occur in less than 2% of all pancreatic tumors (1). However, the incidence of PNENs has been reported to have increased dramatically over the past few decades, resulting in 0.8/100,000 per year (2). Clinically, most PNENs are episodic, occurring between the 40th and 60th years (3). PNENs are classified into functional and non-functional according to the presence of other endocrine-related symptoms (4). Of PNENs, 60%–90% are non-functional, causing local symptoms or accidental diagnosis due to mass effects, while 30% of cases are functional, whose symptoms are associated with excessive secretion of hormones and amines (5).

Symptoms of PNENs include abdominal pain, weight loss, jaundice, and other common symptoms (6). The liver is the most common metastatic site for PNENs (7). In the case reported here, the patient had chronic hepatitis C, and liver masses were found during his regular follow-up. Preoperative enhanced MRI of the patient’s liver indicated a nodular lesion in the lower right lobe of the liver. Enhanced MRI showed “fast in and fast out”, which was consistent with the imaging findings of primary hepatocellular carcinoma. Therefore, before surgery, the patient was clinically diagnosed with primary liver cancer.

Hepatectomy is the most common treatment for liver malignancies. The histopathological findings of the specimen revealed a synaptophysin-positive and chromogranin-A-positive neuroendocrine neoplasm. At that time, we are more interested in whether the patient has a primary tumor of the pancreas or gastrointestinal tract. But no evidence of a primary tumor was found. The patient recovered well after surgery and was followed up every year. Primary pancreatic mass was observed in 2020. Meanwhile, MRI revealed multiple hepatic metastases. This patient presented with a slow-growing primary tumor of the pancreas with a long survival despite a diffuse tumor burden in the liver. The definition of quiet cancer effectively describes the clinical behavior of cancer (2).

#### Diagnostic Method

The most commonly used test for liver malignancies is enhanced liver CT or MRI. For PNENs, functional imaging of ^68^GA-DOTATOC-PET/CT may be a preferred method (8). Because somatostatin receptors are often overexpressed in PNENs, radiographically labeled somatostatin analogs may provide more valuable diagnostic information. The widely used ^18^F-FDG PET/CT is also an alternative method. In this case, ^18^F-FDG PET/CT examination confirmed pancreatic neuroendocrine tumor with multiple liver metastases in 2020. No transfer of other organs or systems occurred. Percutaneous liver biopsy is an effective way to diagnose liver metastases. Percutaneous liver biopsy not only can distinguish a benign tumor from a malignant tumor but also can provide a basis for finding the primary lesion. It has been reported that endoscopic ultrasound (EUS) puncture biopsy of pancreatic tumors can provide effective diagnostic information for pancreatic tumors (9, 10). In this case, a biopsy of the liver and pancreas was not performed because the patient refused any invasive procedures.

#### Therapeutic Method

Hepatectomy is the most common treatment for resectable malignant tumors of the liver and pancreas. EUS-guided radiofrequency ablation (EUS-RFA) has been used as an alternative treatment for PNENs in recent years (11, 12). Multiple lesions in the liver were not suitable for surgical and ablative treatment, so it was decided that the patient undergo hepatic artery embolization and endocrine therapy after a multidisciplinary consultation (MDT). At the same time, anticoagulation with low-molecular-weight heparin calcium and rivaroxaban was given. Follow-up results showed no change in the size of the pancreatic lesion but a reduction of intrahepatic tumor, which suggests that hepatic artery embolization can quickly reduce the burden of intrahepatic tumors. Octreotide acetate microspheres for injection were used every 4 weeks as maintenance therapy, but surgical resection was not performed because of the multiple lesions in the liver, which were not suitable for surgical resection. The patients were followed up once a month. At present, the patients’ conditions were all in a stable state, achieving the purpose of survival with tumor and improving the quality of life, without any clinical uncomfortable symptoms.

### Portal Vein Thrombosis

Portal vein thrombosis is divided into secondary and primary according to the etiology (13). Tumor invasion or compression into the portal vein, inflammation cascade related to pancreatitis, coagulation factor consumption, endocrine dysfunction, abdominal trauma, changes in platelet function and activity caused by surgery, absence of coagulation factors in the blood system, slow blood flow in the portal venous system, hypercoagulation, and damage to the vascular endothelium caused by drugs (antitumor drugs, contraceptives, and hemostatic drugs) are common thrombotic factors (14).

There has been a link between vein thrombosis and cancer for centuries (15). Although the exact individual incidence of portal vein thrombosis in liver cancer has not been defined, there is evidence that the absolute risk depends on tumor type, degree of tumor load, and chemotherapy agent (16). Based on recent researches, the cancer-specific risk of idiopathic venous thromboembolism is the highest for ovarian, pancreatic, and liver cancers with a downward trend (17). Thromboembolism is a recognized complication of malignancy and has long been diagnosed as a phenomenon occurring simultaneously with recessive malignancy (15, 18). Tumor growth is associated with the development of hypercoagulability. In patients with malignancy, the disease usually presents with abnormal laboratory clotting tests and even non-obvious thrombosis, indicating that fibrin formation and removal are a continuous process (19).

**Figure 4:**
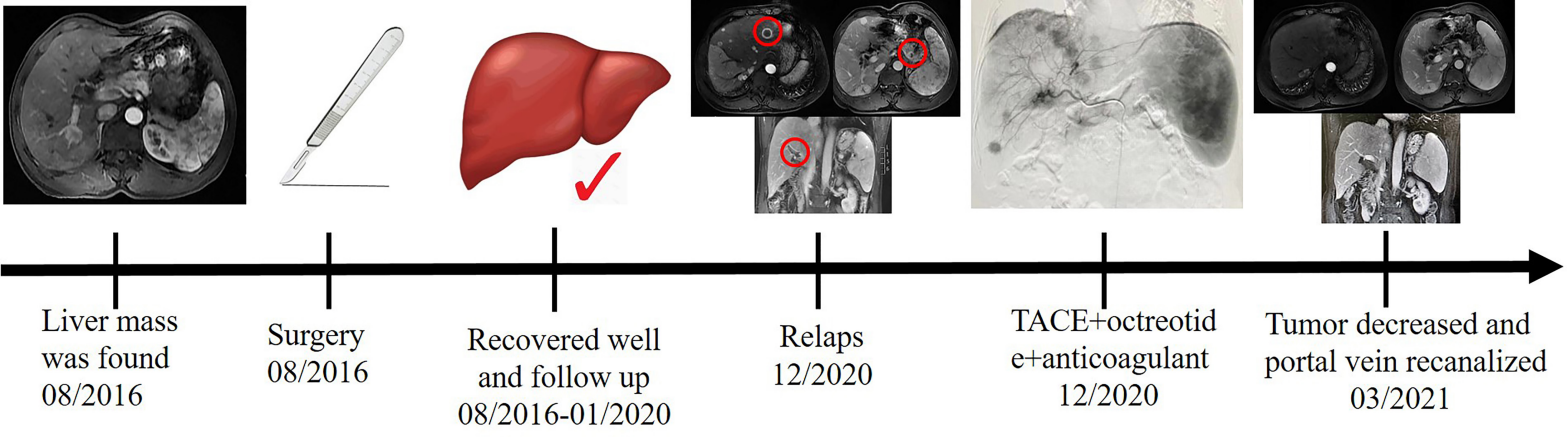
A timeline containing data related to treatment.

Portal vein thrombosis is common in patients with cirrhosis (20). It is necessary to diagnose and manage acute symptomatic portal vein thrombosis in a timely fashion. Failure to detect and treat thrombus can lead to complications of mesenteric ischemia, chronic portal vein cavernous transformation, and portal hypertension (21). In patients with cirrhosis, portal vein thrombosis often develops insidiously and remains undetected until incidental testing (22). Management of portal vein thrombosis in patients with cirrhosis is more controversial (23). However, there are data to support the use of anticoagulants in specific patients (24). After anticoagulation treatment, the patient’s portal vein thrombosis recanalized, and splenic vein thrombosis partially recanalized. Abnormal intrahepatic blood supply was also recovered well. A timeline containing data related to treatment was showed in **Figure 4**. Full-text case reports of patients with pancreatic neoplasms with liver metastases and portal vein thrombosis were listed in **Table 1**.

**Table 1:** Full-text case reports of patients with pancreatic neoplasms with liver metastases and portal vein thrombosis.

	Year	Reference	Sex	Age	Type of pancreatic neoplasm	hepatic metastases	Portal vein tumor thrombus	Portal vein thrombosis	Anticoagulanttherapy

1	2004	Usatoff et al. (25)	F	59	Cystadenocarcinoma of pancreas	No	Yes	No	No
2	2004	Bedirli et al. (26)	F	60	Non-functioning islet cell carcinoma of the pancreas	No	Yes	No	No
3	2005	Nguyen (27)	M	44	Pancreatic neuroendocrine neoplasms	Yes	Yes	No	No
4	2006	Kawakami et al. (28)	M	68	Non-functioning endocrine tumor of the pancreas	No	Yes	No	No
5	2007	Zyromski et al. (29)	M	69	Pancreatic cancer	No	No	Yes	No
6	2008	Roldán-Valadez et al. (30)	M	68	Pancreatic adenocarcinoma	No	No	Yes	No
7	2010	Vadalà et al. (31)	M	74	Serous cystadenocarcinoma of the pancreas	No	Yes	No	No
8	2010	Nara et al. (32)	F	79	Anaplastic carcinoma of the pancreas	No	Yes	No	No
9	2011	Maknia et al. (33)	F	39	Acinar cell carcinoma of the pancreas	No	No	Yes	Yes*
10	2011	Lim et al. (34)	M	37	Pancreatic neuroendocrine neoplasms	Yes	Yes	No	No
11	2011	Tsuchikawa et al. (35)	M	60	Pancreatic endocrine tumors	No	Yes	No	No
12	2012	Reilly et al. (36)	M	23	Pancreatic Ewing’s sarcoma	No	Yes	No	No
13	2014	Machimoto et al. (37)	M	63	Pancreatic cancer	No	Yes	No	No
14	2017	Su et al. (38)	F	54	Pancreatic cancer	No	No	Yes	No
15	2020	Laclau-Lacrouts et al. (39)	M	54	Intraductal papillary mucinous neoplasm (IPMN)	No	Yes	No	No
16	2021	Ishida et al. (40)	M	79	Pancreatic neuroendocrine neoplasms	No	Yes	No	No

^*^Anticoagulant therapy was performed, but the efficacy of portal vein thrombosis was not described.

### Limitation

The patient lost the chance of radical treatment because PNENs were found to be accompanied by multiple liver metastases during follow-up in 2020. Therefore, a biopsy of pancreatic and liver masses was not performed, and the treatment of the current condition was guided by previous medical history and pathological results.

## Conclusions

We reported a rare case of hepatic metastasis from PNEN with portal vein thrombosis who had a favorable outcome with a combination of therapy. The purpose of this report is to review what is known about patients with hepatic metastasis from pancreatic neuroendocrine carcinoma, in particular the exploration of individualized treatment options for patients with portal vein thrombosis.

## Data Availability Statement

The original contributions presented in the study are included in the article, further inquiries can be directed to the corresponding author/s.

## Ethics Statement

The studies involving human participants were reviewed and approved by the Ethics Review Committee of the First Affiliated Hospital of China Medical University. The patients/participants provided their written informed consent to participate in this study. Written informed consent was obtained from the individual(s) for the publication of any potentially identifiable images or data included in this article.

## Author Contributions

YT: conceptualization, data curation, formal analysis, resources, writing—original draft, and writing—review and editing. XQ: methodology, supervision, and writing—review and editing. AA: formal analysis, investigation, and validation. KX: funding acquisition, investigation, and supervision. HZ: funding acquisition and project administration. All authors contributed to the article and approved the submitted version.

## Funding

This study was funded by the National Key Research and Development Program of China (Grant No. XLYC1802098). The corresponding author, HZ, is the chief expert of the project.

## Conflict of Interest

The authors declare that the research was conducted in the absence of any commercial or financial relationships that could be construed as a potential conflict of interest.

## Publisher’s Note

All claims expressed in this article are solely those of the authors and do not necessarily represent those of their affiliated organizations, or those of the publisher, the editors and the reviewers. Any product that may be evaluated in this article, or claim that may be made by its manufacturer, is not guaranteed or endorsed by the publisher.
